# Editorial: Advancement in Cancer Stem Cell Biology and Precision Medicine

**DOI:** 10.3389/fcell.2022.890129

**Published:** 2022-03-25

**Authors:** Nikhil Baban Ghate, Vicky Yamamoto, Eliza Chakraborty

**Affiliations:** ^1^ Department of Biochemistry and Molecular Medicine, Norris Comprehensive Cancer Center, University of Southern California, Los Angeles, CA, United States; ^2^ Society for Brain Mapping and Therapeutics (SBMT), Los Angeles, CA, United States; ^3^ Brain Mapping Foundation (BMF), Los Angeles, CA, United States; ^4^ The USC Caruso Department of Otolaryngology-Head and Neck Surgery, USC Keck School of Medicine, Los Angeles, CA, United States; ^5^ USC-Norris Comprehensive Cancer Center, Los Angeles, CA, United States; ^6^ Department of Biotechnology, DST-FIST Center, Meerut Institute of Engineering and Technology, Meerut, India

**Keywords:** cancer stem cell, metastasis, head and neck cancer, tumor microenvionment, squamous cell carcinama

Cancer Stem Cells (CSCs) are small subpopulation of cells within tumors with capabilities of self-renewal, differentiation, and tumorigenicity. The clinical relevance of CSCs has been strengthened by emerging evidence suggesting that CSCs are thought to cause treatment resistance, recurrence, and metastasis (Yu et al., 2012). Hence, targeting CSCs for understanding the root-cause of tumorigenesis and development of anticancer therapies can be considered as effective strategy to improve cancer outcomes. CSCs can also be targeted for the development of diagnostic biomarkers to develop personalized oncology approaches. For instance, in glioma patients, precision diagnosis and prognostic prediction can be obtained by checking the expression level of ETV2 gene. ETV2 is found to be involved in the invasion, migration, and epithelial-mesenchymal transition process of glioma. The selection of this gene for diagnosis and prognosis purpose was based on stemness subtype-related risk score model and nomogram. To construct this model RNA-seq data were analysed and 86 mRNA expression-based stemness index-related differentially expressed genes were obtained and combined with weighted gene correlation network analysis (Tan et al.). In another study, Ban et al. calculated the stemness index for samples of multiple myeloma and found that mRNA expression-based stemness index (mRNAsi) was an independent prognostic factor of multiple myeloma. The study also built 34-gene based classifier for predicting prognosis and potential strategies for stemness treatment. However, in case of head and neck squamous cell carcinoma where distinct intratumoral and intertumoral heterogeneity has hindered both the identification of specific biomarkers as well as the establishment of targeted therapies. Hence there is an urgent need for finding strategies for personalized therapeutic options in head and neck cancer patients. In one of the reviews, authors discussed the different approaches in implementing three-dimensional (3D) head and neck cancer *in vitro* and *in vivo* tumor models like spheroid, 3D co-culture, bioprinting, CSC-enriched, and patient-derived explant models for the development of stable biomarkers and for therapeutic efficacy testing in clinical studies (Affolter et al.). In these efforts, to establish a new *in vitro* model having potential value on personalized cancer therapies, Dong et al. propose a cell-culture based technique of conditional reprogramming (CR) of laryngeal and hypopharyngeal squamous cell carcinoma. In this model, the patient-derived CR cells could be transformed to xenograft and organoid and they show similar drug responses compared to clinical studies. In another interesting study, authors have found the presence of hematopoietic stem cell (HSC) transcriptome in human fetal kidney cortex for the first time. Previously, this was only observed in zebrafish kidneys. The authors have identified the presence of cells expressing HSC specific marker RUNX1 because it plays a major role in HSC generation (Hwang et al.). Non-coding RNAs (ncRNAs) were initially dubbed as “junk RNAs” but increasing evidence indicates that ncRNAs, such as microRNAs (miRNAs) and long non-coding RNAs (lncRNAs) regulate cancer cell stemness and help maintain CSC populations. In another detailed review on osteosarcoma, authors discussed the role of miR-26a, miR-29b, miR-34a, miR-133a, miR-143, miR-335, miR-382, miR-499a, miR-1247, and let-7d miRNAs in targeting CSCs. They also highlighted the functions of lncRNAs in regulating CSCs in osteosarcoma, such as B4GALT1-AS1, DANCR, DLX6-AS1, FER1L4, HIF2PUT, LINK-A, MALAT1, SOX2-OT, and THOR. The authors believed that targeting these ncRNAs in osteosarcoma might be an effective and novel strategy to eradicate CSCs for osteosarcoma therapy (Liu et al.). Head and neck cancer is one of the cancers in which its 5-years survival has not improved much in the past several decades. Heft-Neal et al. offered a comprehensive review of how the tumor-microenvironment (TME) plays important role in maintaining cancer stem cell niche as well as in treatment resistance. Unlike most reviews on the similar subject, the review also focused on the TME’s involvement in tumor heterogeneity in head and neck cancer, which is thought to be the major cause of treatment resistance. Understanding the mechanisms of how heterogeneity arises could lead to effective personalized cancer therapy.

The review by Man et al. highlighted the importance of hematopoietic stem cell (HSC) niche, which is the microenvironment for the maintenance of stem cells, namely, the cellular components and cytokines in the bone marrow (BM) environment. Understanding the BM niche’s role in maintaining normal HSCs is essential in understanding how blood cancer develops and in developing effective treatments. This review also provided the roles of BM niche in successful HSC transplantation, which is a life-saving procedure for many hematologic diseases, including leukemia as well as potentially effective therapeutic targets for leukemia.

Finally, Lv et al. reviewed mechanisms of liver cancer stem cell (LCSC) maintenance and survival from over 150 + articles. This comprehensive review covers from important signaling pathways, niche composition, the origin of LCSC to new developments in therapeutics targeting LCSCs. Liver cancer, notably hepatocellular carcinoma, is the leading cause of cancer-related deaths worldwide without effective treatments. Many of the important signaling pathways described in the article, including Wnt, STAT3, TGF-b, Notch, Hedgehog, and BMI1 are known to play important roles during embryonic development and normal physiology. The review underscored the similarity between the normal and cancer stem cell and the complexity of CSC biologic property that we still have much to learn.

While some progress has been made in the treatment for leukemia and solid tumors, such as breast cancer, we still have unmet challenges in improving overall survival and outcomes for common cancers, especially when diagnosed late, such as lung, colon, liver, brain, and head and neck cancers. These articles highlight the emerging evidence that stem cell-like or cancer stem cells may play important roles in cancer development, progression, metastasis, and treatment resistance. The next challenge for scientists investigating cancer stem cells is to delineate the differences between normal stem cells and cancer stem cells, which may enable scientists and clinicians to develop more effective cancer diagnostics and therapeutics.
